# Coyotes can do ‘puppy dog eyes’ too: comparing interspecific variation in *Canis* facial expression muscles

**DOI:** 10.1098/rsos.241046

**Published:** 2024-10-02

**Authors:** Patrick Cunningham, Mahita Shankar, Bridgett vonHoldt, Kristin E. Brzeski, Sarah S. Kienle

**Affiliations:** ^1^ Department of Biology, Baylor University, Waco, TX 76707, USA; ^2^ Department of Ecology and Evolutionary Biology, Princeton University, Princeton, NJ 08544, USA; ^3^ College of Forest Resources and Environmental Science, Michigan Technological University, Houghton, MI 49931, USA

**Keywords:** *Canis latrans*, *Canis familiaris*, morphological evolution, AU101, inner brow raise

## Abstract

Facial expressions are critical for non-verbal communication. The *Canis* genus epitomizes the interplay between behaviour and morphology in the evolution of non-verbal communication. Recent work suggests that the levator anguli oculi medialis (LAOM) muscle is unique to dogs (*Canis familiaris*) within the *Canis* genus and evolved due to domestication. The LAOM raises the inner eyebrows, resulting in the ‘puppy dog eyes’ expression. Here, we test whether the LAOM is a derived trait in dogs by (i) examining the facial expression muscles of a closely related and ancestral wild *Canis* species, the coyote (*C. latrans*) and (ii) comparing our results with other *Canis* and canid taxa. We discover that coyotes have a well-developed LAOM like dogs, which differs from the modified/absent LAOM in grey wolves. Our findings challenge the hypothesis that the LAOM developed due to domestication. We suggest that the LAOM is a basal trait that was lost in grey wolves. Additionally, we find inter- and intraspecific variations in the size of the muscles of the outer ear, forehead, lips and rostrum, indicating potential adaptations related to sensory perception, communication and individual-level functional variations within canids. Together, this research expands our knowledge of facial expressions, their evolution and their role in communication.

## 1. Introduction

Facial expressions are a key component of non-verbal communication used to convey emotional state, social status and intention [1–3]. In 1872, Darwin highlighted the need to compare inter- and intra-specific variations in facial expressions to understand the evolution of different types of communication [4]. Using facial expressions to communicate with others involves moving the underlying muscles of facial expression [1–3]. Animals that routinely use facial expressions for information sharing often have a greater number and more robust facial expression muscles than animals lacking this ability [5–8]. Further study of facial expression muscles will provide a greater ability to test hypotheses regarding the evolution of musculoskeletal traits and communication.

The dog–human relationship offers valuable insights into how facial expressions are associated with behavioural and morphological evolution [1,9,10]. Dogs (*Canis familiaris*) were domesticated from wolf-like *Canis* ancestors approximately 33 000 years ago [11]. During the domestication process, dogs evolved unique adaptations associated with their relationship to humans, including more fluid reproductive behaviour, paedomorphic facial features and hyper-social behaviour [12–14]. Furthermore, the facial muscles of dogs have been well described, in direct contrast to most mammals [15]. As a result, studies have demonstrated that dogs have many robust facial muscles that generate expressions associated with both affiliative (e.g. initiating play) and agonistic (e.g. preceding aggressive behaviour) contexts [16–21].

The dog’s ability to make ‘puppy dog eyes’ has received a lot of attention over the last decade. The ‘puppy dog eyes’ expression is controlled by the levator anguli occuli medialis (LAOM) muscle, which originates on the frontal bone and attaches to the medial skin of the eyebrow and acts to raise the inner eyebrow [13,22–24]. By raising the inner brows, the height of the orbital cavity appears to increase, which makes the eyes seem larger. Together these two actions result in the inner brow raiser facial expression, creating the iconic ‘puppy dog eyes’ [13]. Therefore, when species like dogs have the LAOM, it suggests the species can create the inner brow raiser expression by contracting this muscle [13,22,25].

**Figure 1.**
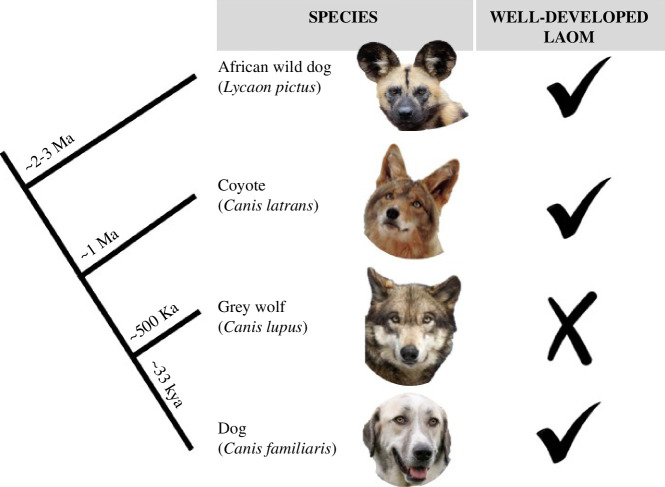
Phylogeny showing the relationship of the four canid species in which the levator anguli oculi medialis (LAOM) has been studied. A well-developed LAOM has been documented in African wild dogs [25,26], coyotes (this study) and dogs [15,22], but is modified/absent in grey wolves [22]. The phylogeny includes the estimated divergence times for each species. The African wild dog (*Lycaon pictus*) estimate comes from fossils that indicate a mid-Pleistocene origin in Africa with possible earlier proliferation approximately 2–3 Ma [27,28]. The coyote (*Canis latrans*) speciated around approximately 1 Ma [29]. The grey wolf estimate is based on a combination of fossil and genetic evidence suggesting a divergence approximately 500 000 years ago in Eurasia [30–38]. After the last glacial maximum (25 000 years ago), dogs and wolves had already diverged, and wolves underwent a severe bottleneck and subsequent radiation resulting in contemporary wolf lineages [30,38,39]. Dogs diverged from their wild counterparts approximately 33 000 years ago, marking the beginning of domestication [11,40]. African wild dog photo by Mathias Appel, CC0; coyote photo by Shreve Stockton; grey wolf photo by Monty Sloan.

Some researchers have suggested that the inner brow raiser expression in the *Canis* genus evolved for non-verbal communication between humans and dogs [13,22,23]. Kaminski *et al*. [22], for example, showed that dogs initiate the inner brow raiser more frequently when humans were attentive as opposed to inattentive. Another study found that dogs that perform the inner brow raiser are adopted from animal shelters more quickly, suggesting that humans are particularly drawn to dogs with this expression [13]. Similarly, a recent dissection-based study compared the facial expression muscles between dogs and grey wolves (*C. lupus*), their sister taxon [22]. Kaminski *et al*. [22] found that dogs have a well-developed LAOM. In contrast, the LAOM in grey wolves is described as ‘absent’ or ‘never present’, as well as ‘typically reduced’ or ‘appeared as a small tendon incompletely separated from the orbicularis oculi muscle’ ([22]; figure 1; based on these variable descriptions, we refer to the LAOM in grey wolves hereafter as modified/absent). Together these studies have led to the hypothesis that human-driven selection for the inner brow raiser during the dog domestication process resulted in the well-developed LAOM in dogs compared with its loss in their wild counterparts (i.e. grey wolves [22]).

In contrast, other researchers suggest that the inner brow raiser expression serves a more basal function associated with eye movements [24]. Bremhorst *et al*. [24], for example, suggested the inner brow raiser expression in dogs is more related to eye movements than interspecific communication. Specifically, they compared the facial expressions of dogs when an attentive human provided a food reward to when no human was present and a food reward was provided by an automated device [24]. They found that dogs exhibited the inner brow raiser more in the absence of a human and noted that the expression was strongly associated with eye movement, occurring independent of eye movement only 6% of the time [24]. Recently, a study documented the LAOM in African wild dogs (*Lycaon pictus*; [25,26]), an ancestral canid species; the muscle has also been described in other carnivores (e.g. servals, tigers [41];) and ungulates (e.g. camels, horses, bowhead whales [42–44]). Given its presence across these diverse mammals, the LAOM may have a more basal function than communication through ocular expression.

To determine the evolutionary polarity of a trait (i.e. the direction of evolutionary change in a trait [45];), like the LAOM, requires establishing whether the trait is ancestral or derived in a given clade [45]. However, it is difficult to compare evolutionary polarity between two sister taxa with an unknown evolutionary history (like dogs and grey wolves) for multiple reasons. First, the evolutionary history of dogs and grey wolves is complex. Grey wolves are the closest living relative of domestic dogs [46], but the evolutionary relationship between grey wolves and dogs is still debated. For example, Kaminski *et al*. [22], assumed that dogs are direct descendants from the same lineage as modern extant grey wolves while other evidence suggests dogs were domesticated multiple times in different regions, potentially involving several wild *Canis* populations and possibly unknown extinct wolf lineages [47–52]. This complexity makes it challenging to determine whether the last common ancestor of the dog is directly comparable to extant grey wolves, which complicates the assessment of whether the LAOM is an ancestral or derived trait [45]. Second, *Canis* species can hybridize and produce fertile offspring [53,54]. As a result, there are several regions of genetic admixture in North America *Canis* species that results in populations with varying proportions of ancestry from the coyote (*C. latrans*), grey wolf, dog, red wolf (*C. rufus*) and eastern wolf (*C. lycaon*) [54–60]. Therefore, variability in the presence and size of the LAOM between dogs and grey wolves may be the result of genetic contributions from other *Canis* taxa. To examine evolutionary polarity for the LAOM, we need to compare the muscle morphology of multiple species in the *Canis* genus, including both ancestral and modern species, to evaluate whether the trait is more primitive or derived.

Within the *Canis* genus, facial expression muscles have only been described for dogs and grey wolves [15,22]. The lack of data for other *Canis* species hinders our ability to resolve the role of the LAOM in the evolution of the human–dog relationship. For example, if the LAOM is present in coyotes, an ancestral *Canis* species closely related to the grey wolf and dog (46,51,61; figure 1), this would challenge the notion of an evolutionary polarity in the presence or absence of the LAOM between grey wolves and dogs [45] and suggest that the LAOM was present in the common ancestor of coyotes, grey wolves and domestic dogs.

The overarching goals of this study are threefold: (i) examine the facial expression muscles, and LAOM in particular, in the coyote and dog; (ii) compare these findings with previously published accounts of grey wolf facial muscles [22] to explore the extent to which the muscles of facial expression vary between these species; and (iii) compare intraspecific variation in the muscles of facial expression within coyotes. We hypothesized that coyotes will have a well-developed LAOM as the LAOM is present in an ancestral canid (the African wild dog) and a more recently diverged canid (the dog). Together, our results will contribute to a broader understanding of the evolution of musculoskeletal anatomy in mammals and provide insights into the complex interplay between behaviour and morphology.

## 2. Methods

### 2.1. Sample collection

We opportunistically collected 10 coyotes (five males, three females and two unknown sex) and one dog (male, Australian cattle dog) to examine their muscles of facial expression under Texas Parks and Wildlife scientific collections permit number SPR-1221-200 (table 1). The dog was included for comparison and to corroborate prior descriptions of dog facial expression muscles [15]. All but one of our coyote specimens (BU008) were collected in McClennan County and Galveston County, Texas. BU008 was donated by a sportsman in Fayette County, Texas. The dog was donated by the Central Texas Humane Society in Waco, Texas. All specimens were adults based on age estimates from tooth wear [62]. Prior to the dissection, the head was separated from the body at the base of the occipital condyle. We took a small tissue sample from the tongue, ear or temporalis for genetic analysis. All specimens were in good condition (i.e. fresh dead with little to no muscle decomposition) and kept frozen in a −20℃ freezer until dissection.

**Table 1.** Demographic information for the coyotes and the dog in this study. (Abbreviations: F, female; M, male; NA, not analysed.)

specimen ID	species	sex	age (years)^a^	location	red wolf ancestry proportion
BU002	*C. familiaris*	M	1–2	McLennan County, TX	NA
BU004	*C. latrans*	M	1–2	Galveston Island, TX	0.66
BU005	*C. latrans*	F	1–2	Galveston Island, TX	NA
BU006	*C. latrans*	M	1–2	Galveston Island, TX	NA
BU007	*C. latrans*	M	1–2	Galveston Island, TX	NA
BU008	*C. latrans*	M	2–4	Fayette County, TX	NA
BU014	*C. latrans*	F	>6	McLennan County, TX	0.21
BU016	*C. latrans*	F	1–2	McLennan County, TX	0.21
BU017	*C. latrans*	M	1–2	Mclennan County, TX	NA
BU019	*C. latrans*	NA	2–4	Galveston Island, TX	NA
BU020	*C. latrans*	NA	1–2	TX (county unknown)	0.68

^a^ Age classes estimated from tooth wear based on Gipson *et al*. [62].

### 2.2. Dissections

Specimens were dissected fresh without any chemical fixation. Prior to beginning each dissection, we took scaled photographs [63] of the dorsal, ventral and lateral sides of the head. For roadkill specimens, we noted any obvious fractures of the cranium or mandible that may have altered the shape of the superficial musculature. We did not include any specimens where the skull or musculature was determined to be in poor condition. We started each dissection with a rostro-caudal incision from the base of the nose pad to the occipital protuberance. The skin was separated from the superficial layer of muscle on the left lateral aspect of the head to allow for examination of the superficial muscles of facial expression. Next, we removed the superficial musculature to reveal the deeper facial musculature. We inferred the action of each muscle from the fibre direction and the location of the origin and insertion sites. For each muscle, we took scaled photographs, described the muscle insertion, origin, fibre direction and action, and noted any interspecific and intraspecific variation in musculature.

We documented the presence, absence and relative size of each muscle in the coyotes and dogs that we dissected. We referred to Kaminski *et al*. [22] for grey wolf anatomy, and we verified our dog findings with reference to Evans & de Lahunta [15]; this enabled comparisons among the three taxa (table 2).

**Table 2.** Variation in the presence, reduction or absence of the muscles of facial expression between coyotes, grey wolves and domestic dogs. Grey wolf data come from Kaminski *et al*. [22]. Dog data come from this study and Evans & de Lahunta [15].

muscle	coyote	grey wolf	dog
platysma	present	present	present
sphincter colli profundus	smaller^a^	present	present
orbicularis oris	present	present	present
zygomaticus	present	present	present
levator labii superioris	present	present	present
caninus	present	present	present
mentalis	present	present	present
levator nasolabialis	present	present	present
orbicularis oculi	present	present	present
retractor anguli oculi lateralis	present	modified/absent^a^	present
levator anguli oculi medialis	present	modified/absent^a^	present
occipitalis	present	present	present
frontalis	smaller^a^	present	present
interscutularis	smaller^a^	present	present
parietoscutularis	present	present	present
parietoauricularis	present	present	present
cervicoscutularis	present	present	present
middle cervicoauricularis	present	present	present

^a^ Indicates differences in a muscle between the three species.

### 2.3. Genetic analysis

We genotyped the coyote specimens to account for the possible influence of dog ancestry on craniofacial morphology. We collected muscle tissue and obtained high molecular weight genomic DNA using the DNeasy Blood and Tissue Kit (Qiagen) following the manufacturer’s protocol. We quantified DNA concentration using the Qubit 2.0 fluorometer system and prepared genomic libraries for RAPTURE RADseq following a modified protocol [64]. Briefly, we used the *Sbf1* restriction enzyme to digest 75 ng of genomic DNA to which we ligated a unique 8 bp barcoded biotinylated adapters. These barcodes allowed the pooling of equal amounts of DNA. Once pooled, we randomly sheared DNA to 400 bp in a Covaris LE220. We used a Dynabeads M-280 streptavidin binding assay to enrich the pools for adapter ligated fragments, and we then retained fragments 300–400 bp in size using Agencourt AMPure XP magnetic beads. We prepared these enriched libraries for Illumina NovaSeq 2 × 150 nt sequencing at Princeton University’s Lewis Sigler Genomics Institute core facility using the NEBnext Ultra II DNA Library Prep Kit. Once we received the raw sequence data, we retained reads that contained the unique barcode and the remnant *SbfI* cut site using *STACKS* v. 2. We demultiplexed the pools using a 2 bp mismatch threshold for barcode rescue in the *process_radtags* module followed by filtering to keep reads with a minimum quality score of 10 and removing PCR duplicates with the *clone*_*filter* module. We mapped the cleaned and processed reads to the reference dog genome CanFam3.1 assembly [65] using BWA-mem [66]. We subsequently filtered mapped reads to retain those that had MAPQ > 20 and converted the data to bam format in *Samtools* v. 0.1.18 [67]. We included RADseq data from canids previously published (coyotes = 39, grey wolves = 19, eastern wolves = 10, captive red wolves = 10; electronic supplementary material, table S1). These additional samples were previously processed and mapped following the above identical methods and thresholds.

We discovered single nucleotide polymorphisms (SNPs) and followed the recommended pipeline for the *gstacks* and *populations* modules in *STACKS* v. 2 [68,69]. We increased the minimum significance threshold and used the marukilow model (flags --*vt-alpha* and --*gt-alpha*, *p* = 0.01). We opted against using the *populations* flag --*write_single_snp* as ancestry inference is best with high-density data. We excluded singleton and private doubleton alleles, removed loci with more than 90% missing data across all samples and removed individuals with more than 20% missing data with *VCFtools* v. 0.1.17 [70]. We employed a minor allele frequency filtering (MAF < 3%) in *PLINK* v. 1.90b3i [71]. We conducted unsupervised clustering of samples using *flashPCA,* we constructed a ‘*statistically neutral and unlinked*’ dataset of SNPs by additional filtering to remove loci within 50-SNP windows that exceeded genotype correlations of 0.5 (with the *PLINK* argument *--indep-pairwise* 50 5 0.5) and deviated from HWE (--*hwe* 0.001).

### 2.4. Statistical analyses

We measured the surface area of each muscle from scaled photographs in ImageJ (v. 1.54e, NIH). Additionally, we measured the surface area of the entire head prior to the removal of soft tissue using scaled photographs. We calculated the muscle-to-skull area ratio (MSR) of each muscle by dividing the surface area of the muscle by the surface area of the head [5,72,73]. This method allows for comparison of muscle size between specimens, while standardizing based on the overall size of each individual [5]. We also calculated the CV (s.d./mean) of each MSR to evaluate intraspecific variability [5,74,75]. A CV close to 0 indicates low variation in MSR for a given muscle, while a CV close to 1 indicates high variation in MSR for that muscle. We then calculated the mean CV for each muscle and considered a muscle to show high or low intraspecific variation relative to the other muscles of facial expression if it exhibited a CV more than 1 s.d. above or below the average.

## 3. Results

**Figure 2.**
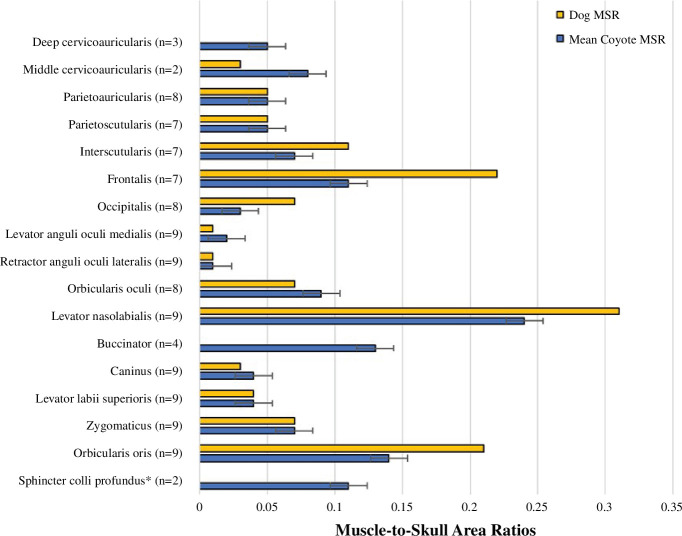
Mean muscle-to-skull area ratios (MSRs), their standard deviations and coyote sample size for each craniofacial muscle measured in this study. When data were absent for the dog, the muscle was still present but measurements were not taken because portions of the muscle were destroyed during the process of reflecting the superficial structures.

**Figure 3.**
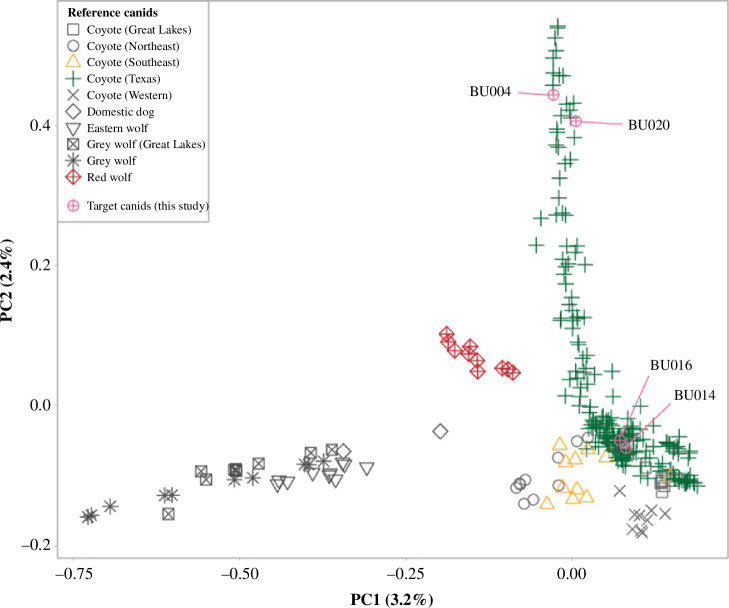
Unsupervised clustering analysis of 295 canids genotyped at 62 729 unlinked and statistically neutral SNPs. Our target canids are indicated by sample IDs.

We found interspecific variation in the facial expression muscles of coyotes, dogs and previous accounts of grey wolves [22], including the presence of a well-developed LAOM in coyotes. We documented intraspecific variation in coyotes’ facial muscle morphology (figure 2). We determined canid ancestry proportions for four coyote specimens (table 1) and did not detect any significant dog ancestry (figure 3); this indicates that dog ancestry is not driving inter- and intra-specific variability in the craniofacial musculature of the specimens in this study.

### 3.1. Interspecific comparison

We identified 18 muscles of facial expression in the coyote and dog (table 2). While most coyote facial expression muscles showed little to no differences in origin, insertion and fibre direction from dogs and previously published data for grey wolves [22], five facial expression muscles show interspecific variability. Specifically, the sphincter colli profundus, frontalis and interscutularis are significantly smaller in coyotes compared with dogs (table 2). While coyotes have a well-developed LAOM and retractor anguli oculi lateralis (RAOL), like dogs, this differs from previous descriptions of grey wolf anatomy [22]. In §§3.1.1–3.1.5, we describe these five muscles in coyotes and describe how this morphology differs across *Canis* taxa.

#### 3.1.1. Sphincter colli profundus

In coyotes, the sphincter colli profundus consists of thin muscle strands that fan across the lateral sides of the head and jaw and branch underneath the ventral side of the head. The muscle is differentiated into (i) the sphincter colli profundus pars palpebralis and (ii) the sphincter colli profundus pars intermedius. The sphincter colli profundus pars palpebralis originates under the eye, where its origin is blended with the orbicularis oculi. The muscle branches ventro-caudally over the zygomaticus before plunging deep into the platysma. Muscle fibres insert into the skin and blend with the platysma to tense and move the skin under the eye and caudal to the mouth. The sphincter colli profundus pars intermedius originates on the scutiform cartilage by the ear and fans ventrally towards the median raphe of the neck. The muscle eventually fuses with fascicles of the sphincter colli profundus pars intermedius from the other side of the head. As the sphincter colli profundus fans out, some fibres run ventral and parallel to the zygomaticus, while others spread ventrally over the masseter, deep to the platysma. Fibres of the sphincter colli profundus adhere tightly to the platysma and act with the platysma to tense and move the ventral and lateral skin of the neck.

In coyotes, both portions of the sphincter colli profundus were smaller compared with dogs. It was often difficult to reflect the coyote’s platysma while maintaining the integrity of the sphincter colli profundus; this was due to the small size of the sphincter colli profundus and its tight adhesion to the platysma. In dogs, both parts of the sphincter colli profundus were visible after reflecting the platysma and were tightly attached to deeper facial expression muscles.

#### 3.1.2. Frontalis

**Figure 4.**
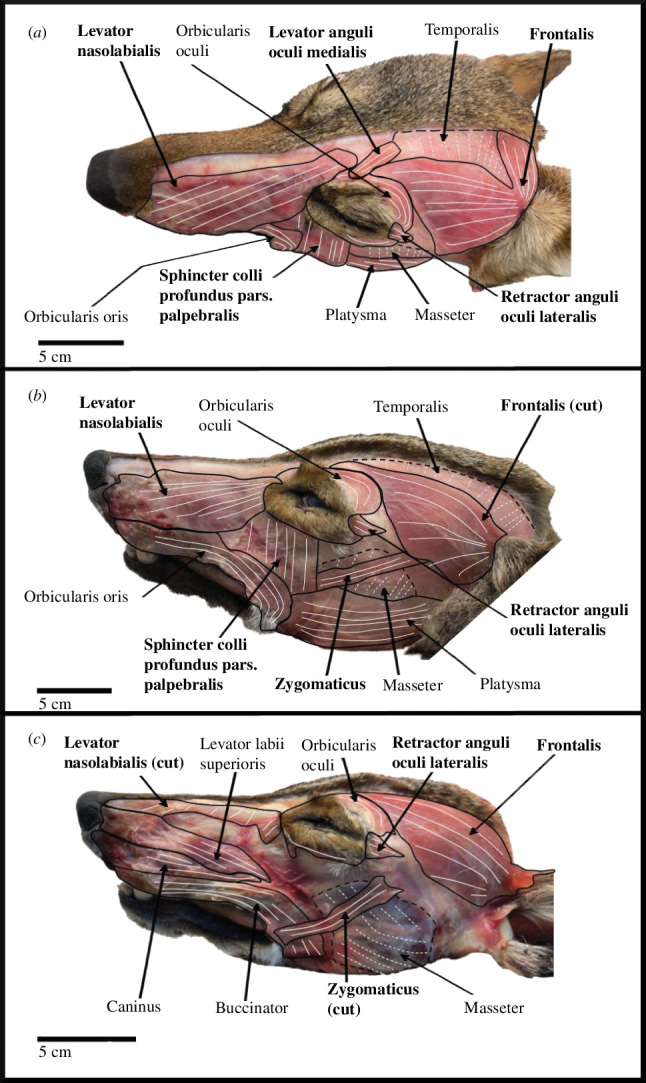
Superficial coyote facial expression muscles are shown in dorsal (*a*) and lateral (*b*) view. Deep facial expression muscles are shown in lateral view (*c*) Fibre directions are shown in white. Dashed lines indicate a deeper muscle. Note the well-developed levator anguli oculi medialis and retractor anguli oculi lateralis, and the faint medial fibres of the frontalis. Bold font indicates a muscle that exhibited inter- and/or intra-specific variation.

In coyotes, the frontalis is a large muscle superficial to the temporalis on the dorsal portion of the cranium. There are two sections of the frontalis: (i) a conspicuous, large, flat, triangular section located between the ear and eye on the lateral aspect of the dorsal portion of the cranium and (ii) a thin, flat section that runs medially between both ears rostral and parallel to the interscutularis. The frontalis originates on the rostral border of the scutiform cartilage, on the nasofrontal fascia and on the zygomatic process. Muscle fibres of the triangular section run caudo-rostrally from the origin along the scutiform cartilage and zygomatic process. The thin, flat section runs laterally across the scalp and merges with the opposite section of the muscle rostral to the interscutularis (figure 4). The frontalis acts to pull the eyebrows towards the ears and wrinkle the skin of the forehead.

In both coyotes and dogs, the triangular portion of the frontalis is well developed, but the portion of the frontalis that runs medially across the scalp is smaller in thickness in coyotes compared with dogs. In coyotes, the lateral portion of the frontalis is so thin that you can see the striations of the temporalis through it, which was not the case for the dog.

#### 3.1.3. Interscutularis

In coyotes, the interscutularis is a thin rectangular sheet of translucent muscle fibres that spans from one scutiform cartilage to the other (figure 4). The muscle is so thin that the occipitalis and cervicoscutularis are often visible through it. The interscutularis originates on the scutiform cartilage immediately caudal to the lateral portion of the frontalis. The muscle fibres run medially from the origin, cover the caudal sagittal crest and occipital protuberance, and insert in the opposite scutiform cartilage. The interscutularis is superficial to the temporalis and occipitalis on the dorso-caudal portion of the cranium, covers the occipitalis and cervicoscutularis and is bordered rostrally by the frontalis. The muscle aids in stabilizing the scutiform cartilage and pulls the scutiform cartilage towards the midline, lifting the ear. In coyotes, the interscutularis is much smaller compared with dogs. In dogs, the interscutularis is more robust; it also fully covers and obscures the deeper muscles (e.g. occipitalis, cervicoscutularis) from view.

#### 3.1.4. Levator anguli oculi medialis

In coyotes, the LAOM is a small, superficial facial muscle. The muscle originates along the median line of the frontal bone from the nasofrontal fascia. The LAOM passes over the orbicularis oculi dorsally to attach to the medial half of the superior eyelid (figure 4). The muscle raises the inner eyebrow medially, resulting in the inner brow raiser expression. The LAOM was present as a distinct, well-developed muscle in every coyote specimen in this study. The coyote’s LAOM is similar in size and orientation to that of the dog.

#### 3.1.5. Retractor anguli oculi lateralis

The RAOL is a small muscle located at the lateral corner of the eye. The muscle originates on the robust facia of the temporalis and extends rostrally to the lateral corner of the eye. The RAOL is partially covered by the orbicularis oculi as it reaches its insertion on the lateral corner of the eyelid. This muscle acts to help squint the eye by pulling the eyelid towards the ears slightly. The coyote’s RAOL is similar in size and orientation to that of the dog.

### 3.2. Intraspecific comparison

In coyotes, five muscles—the LAOM, zygomaticus, levator nasolabialis, parietoscutularis and parietoauricularis—showed high levels of intraspecific variation in their origin, insertion and/or fibre direction (CV > 0.49; electronic supplementary material, table S2). In contrast, three muscles—the orbicularis oris, buccinator and frontalis muscles—exhibited low intraspecific variation (CV < 0.23). In §§3.2.1–3.2.5, we describe intraspecific variation in the five muscles with high variability.

#### 3.2.1. Zygomaticus

In coyotes, the zygomaticus is a long, conspicuous muscle located on the lateral aspect of the face. The muscle originates from the scutiform cartilage and extends to its insertion at the corner of the mouth (figure 4). The fibres run diagonally and caudo-rostrally from origin to insertion. The fibres bunch together to form a tight band along most of the muscle and then fan out slightly at its insertion. The zygomaticus acts on the corner of the lips, pulling the lips laterally. Portions of the zygomaticus run deep to the frontalis, sphincter colli profundus pars palpebralis, platysma and orbicularis oris. The zygomaticus is superficial to the temporalis, masseter and buccinator. The muscle is bordered ventrally by some strands of the sphincter colli profundus pars intermedia.

The zygomaticus has a high CV (0.53; electronic supplementary material, table S2). The extent to which the zygomaticus fans at its insertion on the corner of the mouth varies slightly between individuals. In some specimens, fibres of the zygomaticus stay fairly concentrated as they near the insertion, whereas others exhibit a more pronounced fan shape as they near the corner of the mouth.

#### 3.2.2. Levator nasolabialis

The levator nasolabialis is the most superficial muscle of the rostrum (figure 4). The muscle covers the dorsal and lateral sides of the rostrum and is superficial to the caninus and levator labii superioris. The levator nasolabialis originates between the orbits on the nasofrontal fascia. The muscle inserts along the superior lip and nose, deep to the orbicularis oris and caudally on the buccinator. The muscle fibres run diagonally from the midline to the lip in a dorsoventral and caudo-rostral fashion. The levator nasolabialis raises the lip and pulls the folds of the nostril.

The levator nasolabialis had a high CV (0.50, electronic supplementary material, table S2). We note that the muscle varied in the degree to which it covered the levator labii superioris. In some specimens, the levator nasolabialis covers the insertion of the levator labii superioris at the external nares, while in others the insertion of the levator labii superioris is visible without reflecting the levator nasolabialis.

#### 3.2.3. Levator anguli oculi medialis (LAOM)

The LAOM (described in §3.1.4 of *Interspecific variation* above) is well developed in all coyote specimens (figure 4). There are some intraspecific differences reflected by a high CV (0.53; electronic supplementary material, table S2). While the LAOM is similar in shape between specimens, the small size of this muscle means that the slight changes in the overall muscle size lead to high variability.

#### 3.2.4. Parietoscutularis

**Figure 5.**
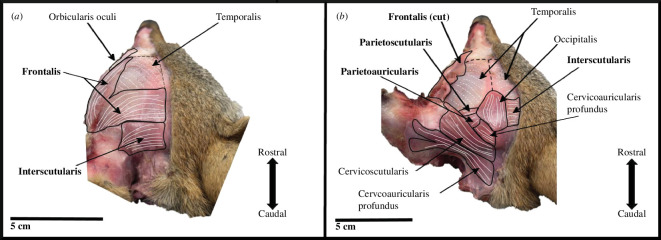
Superficial (*a*) and deep (*b*) coyote craniofacial muscles are shown in caudal view. Note the occipitalis is visible through the faint medial fibres of the frontalis and interscutularis. Bold font indicates a muscle that exhibited inter- and/or intra-specific variation.

The parietoscutularis is a deep muscle of the external ear and is located on the dorsocaudal portion of the braincase (figure 5). The muscle originates on the sagittal crest rostral to the occipital protuberance and inserts on the deep surface of the scutiform cartilage, rostral to the parietoauricularis. The parietoscutularis is deep to the cervicoauricularis superficialis and cervicoscutularis but superficial to the temporalis. At its insertion and along its caudal border, the muscle is fused with the parietoauricularis and the two muscles split as they approach the scutiform cartilage.

The parietoscutularis had a high CV (0.52; electronic supplementary material, table S2). The extent to which the parietoscutularis covers the caudal and dorsal border of the scutiform cartilage varies. In some coyotes, the insertion of the parietoscutularis appears to cover more of the scutiform cartilage, covering almost half the cartilage. In others, the insertion is less robust. Some specimens maintain fusion between the parietoscutularis and parietoauricularis until closer to the scutiform cartilage compared with others. In some specimens, the parietoscutularis and parietoauricularis maintain fusion until very close to the scutiform cartilage. The two muscles finally split; the parietoscutularis inserts on the scutiform cartilage, while the parietoauricularis is attached to the ear itself. In some coyote specimens, this split between the parietoscutularis and parietoauricularis happened prior to reaching the scutiform cartilage.

#### 3.2.5. Parietoauricularis

The parietoauricularis (figure 5) is similar to the parietoscutularis. The two muscles were fused in every coyote specimen and only separated near their respective insertions. The parietoauricularis originates on the sagittal crest caudal to the parietoscutularis and rostral to the occipital protuberance. Along its rostral border, the parietoauricularis is fused with the parietoscutularis, and the two muscles split as they approach the scutiform cartilage. The parietoauricularis inserts on the ear, rostral to the scutiform cartilage. The parietoauricularis is also deep to the cervicoauricularis superficialis and cervicoscutularis but superficial to the temporalis. The parietoauricularis has a high CV (0.50; electronic supplementary material, table S2). Between individuals, the muscle varies at its insertion. Some coyotes have a longer attachment site on the ear than others.

### 3.3. Genomic ancestry

We sequenced 298 canids with an average depth of 15-fold coverage. We built 8 502 624 loci comprising 828 860 455 forward reads and 783 872 862 matching paired-end reads with a mean insert length of 362 (s.d. = 107.8) nucleotides. We obtained an average of 15-fold (s.d. = 11.3) effective per-sample sequence coverage. After removing three samples due to high missingness we retained 86 187 SNPs genotyped across 295 canids after filtering for minor alleles and missingness (electronic supplementary material, table S3). We further identified 62 729 SNPs that were statistically neutral and unlinked. Using an unsupervised clustering method, we found that our study canids spanned a gradient of variation across principal component 2 (PC2). We find two specimens (BU014 and BU016) cluster with Texas and southeastern coyotes at lower PC2 values, while the positive values of BU004 and BU020 exceed the genetic variation observed in red wolves, which are notably distinct from the other reference canids along PC2 (figure 3). All four specimens that we successfully genotyped had mixed ancestry ranging from 21–68% red wolf ancestry, with the remaining ancestry to reference coyotes (table 1).

## 4. Discussion

This is the first study to examine the muscular morphology of coyotes. While the facial expression muscles of coyotes are broadly similar to dogs and grey wolves [15,22], there are some notable exceptions. Specifically, we discover that coyotes have a well-developed LAOM, the muscle responsible for raising the inner brows. Therefore, we find that coyotes can make the inner brow raiser expression, or ‘puppy dog eyes’. Coyotes share this trait with dogs. Therefore, both coyotes and dogs differ from grey wolves where the LAOM is modified/absent ([22]; figure 1).

Because the LAOM is present in coyotes, a species basal to both the grey wolf and the dog, this trait is probably primitive [45]. Our study, therefore, challenges the hypothesis that the inner brow raiser expression is a product of the dog domestication process [13,22,23]. The LAOM is probably a shared characteristic across *Canis* species that was lost/modified in grey wolves. Furthermore, the discovery of the LAOM in the African wild dog [25,26], a species basal to the entire *Canis* genus [76,77], suggests that the LAOM may be a shared morphological trait across most canids. While we cannot completely contradict the hypothesis that the dog’s LAOM was derived because of interspecific communication with humans, our findings suggest that the evolution of the LAOM was driven by factors beyond domestication.

Some of our coyote specimens had substantial red wolf ancestry—up to 68%. We predicted that some of our specimens may have some level of red wolf ancestry because several specimens were collected in a recently rediscovered *Canis* admixture zone near the Gulf of Mexico [56,59]. Based on the presence of the LAOM across this coyote–red wolf admixture gradient, we suggest that red wolves may also have a well-developed LAOM, and therefore the ability to make ‘puppy dog eyes’. Future studies will need to include red wolves to confirm this prediction and determine how genetic admixture influences muscular phenotypes. Our findings also suggest that future studies of canid taxa should consider the effect of genetic admixture and ancestry on patterns of inter- and intra-specific musculoskeletal variability.

Both coyotes and dogs have the same 18 facial expression muscles, while grey wolves only have 16 [15,22]. Both the LAOM (or the inner brow raiser) and RAOL (which functions to squint the eyes) are modified/absent in grey wolves [22]. Still, the reason for their absence is unknown. One possible reason is that dogs have a more pronounced orbital angle relative to grey wolves [78]. If the LAOM moves the brow out of the animal’s line of sight, then the LAOM in dogs may have increased in size/robustness over time to increase their field of view compared with grey wolves. Notably, this would not explain the presence of the LAOM in coyotes, which have similar orbital angles to grey wolves [79]. The lack of the LAOM in grey wolves may also be related to ancestry. For example, Kaminski *et al*. [22] included a total of four grey wolf specimens in their study describing the facial expression muscles of grey wolves, and two were from Michigan. The Great Lakes region is an area of significant admixture between *Canis* species [54,57,80]. Admixture events introduce genetic complexities that might contribute to incomplete penetrance of genes associated with the LAOM, and selection pressures specific to grey wolves might explain why these specimens did not exhibit this muscle despite potential coyote ancestry [81,82]. Therefore, it would be useful to compare the LAOM and RAOL between grey wolves of known ancestry, as well as between individuals with different proportions of genetic admixture, to evaluate the degree to which this trait may be taxonomically diagnostic.

Regardless of why the facial expression muscles of grey wolves, dogs and coyotes differ, the presence of the LAOM in coyotes and modification/absence in grey wolves [22] provides the first useful discrete trait in wild *Canis* taxa. Body size and shape have traditionally been used to distinguish between *Canis* species in North America [83–88], yet these taxa exhibit a gradual morphological continuum of body sizes across species. Combined with the prevalence of genetic admixture between species, this morphological continuum has made clear taxonomic identification challenging. As a result, the phylogeny of North American *Canis* species is an area of continued scientific debate [88–93]. Discovery of the LAOM may therefore serve as an important phylogenetic marker that distinguishes these species, and a valuable tool in clarifying the complex phylogenetic relationships among North American *Canis* species. As such, additional research on the LAOM (and other musculoskeletal traits) in other North American *Canis* species could help clarify species identification, reduce ambiguity in taxonomy and enhance our understanding of the relationships within the North American *Canis* genus [87,88,94].

Except for the LAOM and RAOL, grey wolf and dog musculature have been described as directly comparable [22]. Similarly, our study shows that most coyote facial expression muscles are also directly comparable to dogs and grey wolves in shape, origin, insertion and fibre direction, with three exceptions. The sphincter colli profundus (which tenses the skin of the cheek and neck), the frontalis (which wrinkles the skin of the forehead) and the interscutularis (which helps stabilize the ears) were smaller in coyotes compared with dogs and grey wolves. While it is unclear what drives these interspecific differences, the reduction of the interscutularis may facilitate faster ear movements that would aid in locating small mammalian prey in tall grass or dense leaf litter. Moreover, dogs have less keratin in their ears as a result of domestication, leading to the ‘floppy’ appearance of many dog breeds’ ears compared with the stable, upright erect appearance of the ears in wild canid species [95–98]. More keratin in coyote ears may reduce strain on the interscutularis, leading to less hypertrophy of the muscle compared with dogs. Alternatively, the smaller size of these muscles may simply be the result of evolutionary drift or other undetermined environmental factors.

Coyotes also showed intraspecific variation in the relative size of a few of their facial expression muscles: the LAOM (raises the inner eyebrow), zygomaticus (pulls the corner of the mouth dorsocaudally), levator nasolabialis (raises the upper lip), parietoscutularis (assists the interscutularis in stabilizing the scutiform cartilage) and parietoauricularis (pulls the ear towards the midline). For example, the shape of the zygomaticus insertion varied between coyotes. In some coyotes, the zygomaticus fanned as it approached its insertion point at the corner of the mouth; in other individuals, the zygomaticus fibres remained tightly fused as it approached the mouth. Schatz *et al*. [99] explored variations in the facial muscles of dog breeds with drastically different head shapes. After examining boxers, English bulldogs, Boston terriers and collie-mixes they noticed similar variation in the zygomaticus as seen in this study, even between individuals of the same dog breed. It is possible that variability in muscle use over an individual’s lifespan drives intraspecific variation in muscle shape. While muscles are known to change in size as a response to stress (i.e. hypertrophy or atrophy), they are not generally known to change much in shape. However, the shape of the anatomical structure on which the muscle inserts or originates may change (e.g. bone can change slightly in shape as a response to mechanical stress [100], which could result in slight deviations in the shape of the muscle origin or insertion [101–104]. In the case of the zygomaticus, most fanning of the zygomaticus occurred as the muscle neared its insertion at the corner of the mouth. It may be that individual variation in the use of the lips across an individual coyote’s lifespan may result in more fanning of the zygomaticus insertion. The intraspecific variation observed in coyote muscles associated with movement of the lips, brow and ears underscores the intricate relationships between individual behaviour and morphology. Future studies should focus on pairing behavioural data with modern imaging techniques to elucidate relationships between variations in individual behaviour and muscular morphology.

## 5. Conclusions and future considerations

Coyotes, dogs and grey wolves have a similar craniofacial morphology, with a few important differences. Like dogs, coyotes have a well-developed LAOM that gives both species the ability to make ‘puppy dog eyes’. Finding the LAOM in coyotes means that this trait occurs in both modern and basal *Canis* species, suggesting that this trait is probably primitive [45]. This finding indicates that the ability to make ‘puppy dog eyes’ is a shared trait within the *Canis* genus, challenging the assumption that the LAOM was derived as a consequence of domestication in the dog. Additionally, coyotes exhibit other inter- and intra-specific differences in muscles associated with lip, neck and forehead movement, as well as ear stabilization. Here, we included a single dog to corroborate previous descriptions of their musculoskeletal anatomy [15] and provide a direct comparison in MSR values between coyotes and dogs. Even so, we note that dogs are extremely variable in their morphology, as there is a 40-fold difference in size between the smallest and largest breeds [105]. Therefore, the MSR ratios provided for the dog in this study should not be considered representative for all dogs, as these ratios will vary depending on the size and breed of the dog. Regardless, while there is intraspecific variation in these muscles (as exemplified in coyotes), these differences are slight compared with interspecific variation in the presence and absence of entire muscles. We anticipate that future studies of coyotes, dogs and grey wolves, as well as other canids, will help clarify the drivers and consequences of inter- and intra-specific variation in craniofacial morphology on the behaviour and ecology of wild and domesticated canids. Overall, this study emphasizes the significance of evolutionary history and comparative approaches in unravelling the complex relationship between form and function.

## Data Availability

Data relevant to this study are uploaded as supplemental material, with the exception of the complete set of dissection photographs [106]. Dissection photographs are available from Dryad [107].
